# An Unusual Case of Heart Failure: Sometimes When You Hear Hoof Beats You Should Think of Zebras

**DOI:** 10.7759/cureus.20801

**Published:** 2021-12-29

**Authors:** Edward J Butt, Michael C Boyars

**Affiliations:** 1 Internal Medicine, University of Texas Medical Branch, Galveston, USA

**Keywords:** electrocardiogram, echocardiogram, hyponatremia, congestive heart failure, cardiac amyloidosis, amyloidosis

## Abstract

This case describes a 74-year-old male who was hospitalized with hyponatremia and worsening systolic and new diastolic heart failure. Workup showed low voltage QRS complexes on electrocardiogram and new diastolic dysfunction on echocardiogram. Because of this clinical scenario amyloidosis was suspected. ATTR amyloidosis was confirmed without doing an invasive endocardial biopsy by the use of immunofixation studies and Technetium 99 PYPm scan, and abdominal fat pad biopsy. The types and manifestations of amyloidosis in general and cardiac amyloidosis, in particular, are reviewed as well as the diagnostic test available to the clinician to confirm this diagnosis.

## Introduction

Amyloidosis describes a variety of clinical entities caused when one of many precursor proteins with unstable tertiary structure misfolds and aggregates as insoluble amyloid fibrils [1]. These fibrils deposit in the extracellular space, organs, and soft tissues causing dysfunction of that organ. In cardiac amyloidosis these amyloid fibrils deposit in the extracellular space of the heart [2]. The gold standard of diagnosis of cardiac amyloidosis is the endomyocardial biopsy, however, there are now less invasive methods to reliably confirm this diagnosis. We present a patient with worsening heart failure and clinical clues suggesting cardiac amyloidosis. The clinical features of cardiac amyloidosis, differential diagnosis, and the diagnostic tools available to establish this diagnosis are reviewed.

## Case presentation

A 74-year-old male presented from an outside hospital (OSH) for nausea, vomiting, dizziness, and hyponatremia. He had a significant past medical history of paroxysmal atrial fibrillation, CHADSVASC 4, chronic heart failure with a reduced ejection fraction of 35-40%, coronary artery disease with recent left heart catheterization showing non-obstructive coronary artery disease. On the way to the OSH, he was found to be in atrial fibrillation with a rapid ventricular response which spontaneously resolved upon arrival. A chest X-ray was reported to show vascular congestion and cardiomegaly. The patient was given 1L normal saline and transferred to our hospital.

On admission, he complained of paroxysmal palpitations, shortness of breath at rest, worse with exertion, and orthopnea for several weeks. His medication included amiodarone 200 mg three times a week, aspirin 81 mg once a day, carvedilol 6.25 mg twice a day, furosemide 40 mg twice a day, lisinopril 2.5 mg once a day, and spironolactone 25 mg once a day. Significant findings on physical exam were a Body Mass Index of 21, jugular venous distention (JVD) 8 cm above the sternal angle, with bilateral basilar crackles, no abnormal heart sounds, and no lower extremity edema. Vitals included a mean arterial pressure of 79-89 mmHg. Pertinent blood work is shown in Table 1.

**Table 1:** Pertinent blood work

	At Outside Hospital	Admission
Serum Sodium:	121 mEq/L	122 mEq/L
Troponin:	0.030 ng/ml	0.048 ng/ml
International normalized ratio	4.7	
Brain Natriuretic Peptide		8,250 pg/ml
Serum Osmolality		271 mOsm/kg
Urine Osmolality		332 mOsm/kg
Urine Sodium		66 mmol/L

**Figure 1:**
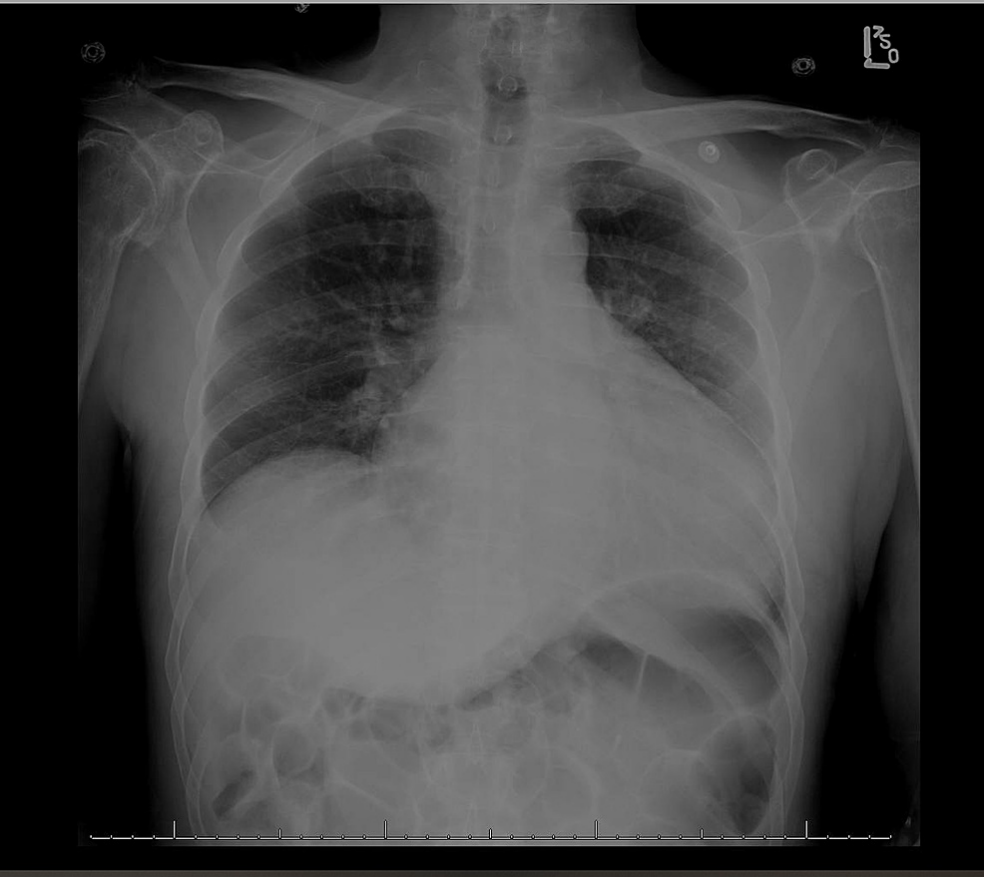
Admission chest X-ray Chest X-ray shows cardiomegaly, small lung fields, and diffuse bilateral infiltrates most consistent with pulmonary edema secondary to congestive heart failure.

Admission chest X-ray is shown in Figure 1.

**Figure 2:**
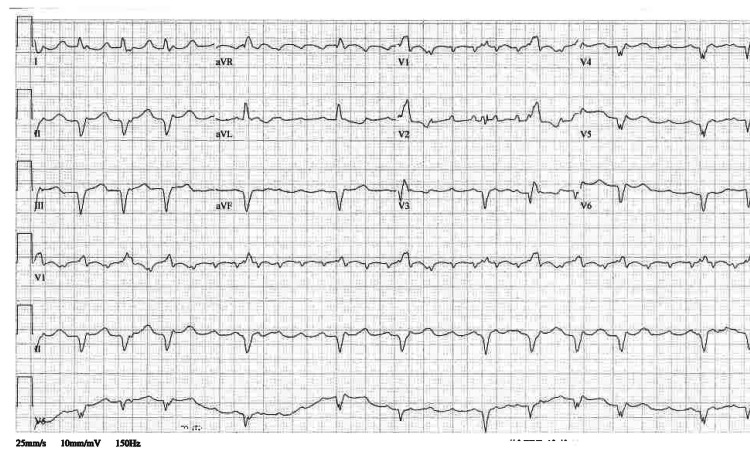
Admission EKG EKG shows low voltage QRS complexes, atrial flutter with variable atrial-ventricular conduction, left axis deviation, right bundle branch block, old inferior and anterolateral infarcts.

The electrocardiogram is shown in Figure 2.

A previous EKG two years prior showed normal sinus rhythm, right bundle branch block, and a first-degree AV block.

A transthoracic echocardiogram (TTE) done on admission showed a left ventricular (LV) ejection fraction (EF) of 25-30% along with severe concentric left ventricular hypertrophy, diastolic dysfunction, a mildly dilated right ventricle, moderate right ventricular (RV) hypertrophy, moderately reduced right ventricular systolic function with an estimated right ventricular systolic pressure (RVSP) of 30-35 mmHg, bi-atrial enlargement, moderate tricuspid regurgitation, mild pulmonic insufficiency, and trace pericardial effusion. A TTE done two years prior showed moderately reduced left ventricular systolic function with an EF of 35-40% with normal-sized atria and ventricles, but no diastolic dysfunction.

During the patient’s hospital stay, aggressive diuresis was initiated with furosemide 40 IV BID. Heart rate control was achieved with metoprolol. In addition, the patient’s previous outpatient medications were restarted with the resolution of the patient’s hypervolemic signs on exam, although sodium levels remained in the low 120’s throughout the patient’s hospital course. The presence and persistence of hyponatremia were thought to be due to worsening systolic and diastolic heart failure. The resultant decreased perfusion of the kidney causes activation of the renin-angiotensin system and anti-diuretic hormone (ADH) secretion. This can mimic the syndrome of inappropriate ADH (SIADH) with increased urine sodium, decreased serum osmolality, and increased urine sodium concentration. SIADH was discounted as the cause for the hyponatremia as it is usually accompanied by a euvolemic state and our patient was clearly hypervolemic. 

Because of the severe left ventricular concentric hypertrophy on echocardiogram, bi-atrial enlargement and low voltage QRS on EKG, ATTR cardiac amyloidosis was suspected. Immunofixation was negative and the Kappa/Lambda ratio was within normal limits. These findings and the absence of organomegaly with normal renal function decreased the suspicion for AL amyloidosis. A Technetium pyrophosphate (99 PYPm) scan demonstrated a Grade 3 on the Perugini scale, showing cardiac uptake with greatly reduced or absent bone signal.

An abdominal fat pad biopsy was performed which detected a peptide profile consistent with ATTR (transthyretin) type amyloid deposition. Liquid chromatography-mass spectrometry did not detect an amino acid sequence abnormality in the transthyretin protein. This finding was consistent with wild-type amyloidosis rather than hereditary transthyretin-associated amyloidosis. Due to the patient’s severe disease and inability to afford tafamidis, the patient opted for hospice. 

## Discussion

Amyloidosis is a disease caused by the deposition of insoluble amyloid fibrils in different tissues of the body, which leads to the abnormal functioning of these tissues [1]. Improper folding and aggregation of proteins cause amyloid formation [1]. Types of amyloidosis include AL (primary) amyloidosis, AA (secondary) amyloidosis, and ATTR (transthyretin) amyloidosis. Classification is based on the type of fibril protein involved.

Among the different types, ATTR and AL amyloidosis can involve the heart, leading to cardiac amyloidosis [2]. Amyloid fibrils infiltrate the ventricles and result in stiffening of the ventricles, leading to restrictive cardiomyopathy [2]. Because of this, cardiac amyloidosis has been known to commonly cause heart failure with preserved ejection fraction (HFpEF), especially in the elderly population. In fact, more than a quarter of patients with HFpEF over the age of 80 are believed to have cardiac amyloidosis [3]. Infiltration of amyloid can also result in conduction abnormalities such as atrial fibrillation, as seen in our patient, and ventricular tachycardia [2].

This case was uncommon in the sense that our patient presented with reduced ejection fraction in addition to diastolic dysfunction. Cardiac amyloidosis has historically been associated with a preserved ejection fraction at the time of diagnosis. In fact, reduced ejection fraction (<40%) is typically only reported in 30% of wild-type ATTR cardiac amyloidosis (ATTR-CA) cases [4,5]. Our patient initially presented with an ejection fraction of 25-30% with an echocardiogram two years prior showing an EF of 35-40%.

Compared to ATTR-CA associated with preserved ejection fraction, patients with ATTR-CA associated with reduced ejection fraction tend to be more symptomatic, New York Heart Association-Functional Class ≥ 3, 61% vs 26%, p<0.001), have mean diastolic dysfunction values that are worse (3 vs 2.15, p<0.01), reduced kidney function (creatinine, 1.63 ± 0.85 vs 1.27 ± 0.55 mg/dl, p<0.01), and higher mortality (35% vs 12%, p=0.002) [6]. In addition, decreases in EF are related to proportional increases in mortality risk [6]. However, there is no difference in biomarkers (troponin and B-type natriuretic peptide [BNP]) or interventricular septal thickness between those with preserved versus reduced EF [6].

On presentation, our patient revealed symptoms and physical exam findings common in heart failures such as orthopnea, elevated JVD, and bilateral crackles. These findings are also commonly found in cardiac amyloidosis patients [7]. Additional manifestations can include macroglossia, nephrotic syndrome, bilateral carpal tunnel syndrome, hepatomegaly, and ascites [7]. Because other diseases such as hypertrophic cardiomyopathy, aortic stenosis, and hypertension can show a similar phenotype, a diagnosis of cardiac amyloidosis can be initially difficult to make [7]. Furthermore, common symptoms of aging such as fatigue, dyspnea, and weakness, can also present in patients with cardiac amyloidosis, making diagnosis challenging [8]. However, multiple diagnostic tools such as EKG, cardiac magnetic resonance, nuclear imaging, and echocardiogram are used to aid in the diagnosis of the disease.

Our patient’s EKG showed low voltage QRS complexes, which is expected in about half of cardiac amyloidosis patients [8]. A pseudoinfarction pattern even without the presence of coronary stenosis is also common [8]. Atrioventricular block, bundle branch blocks, and intraventricular conduction delays are also usually seen on EKG in these patients [8]. Although EKG is a great tool that can be used to suggest a potential diagnosis, EKG findings alone cannot be used to make a definitive diagnosis.

Cardiac magnetic resonance is another modality that can be used. This provides information on the properties of the myocardial tissue. For example, amyloid deposition in the cardiac interstitium results in myocardial injury. These sites of injury act as areas for gadolinium to accumulate, which causes late gadolinium enhancement [9]. This method provides an 80% sensitivity and 94% specificity for diagnosing cardiac amyloidosis [10].

A third way to aid in the diagnosis of cardiac amyloidosis involves nuclear imaging, which is the route that was taken with our patient. This approach uses radiotracers, typically Technetium-99m-pyrophosphate (99mTc-PYP) and Technetium-99m and 3,3-diphosphono-1,2-propanodicarboxylic acid (99mTc-DPD). These nuclear imaging studies show very high specificity and sensitivity, which is why we did not pursue an endomyocardial biopsy to make a diagnosis [11]. Our patient had a high myocardial uptake of 99mTc-PYP, which reflected a poor prognosis [12].

Lastly, an echocardiogram provides many useful clues in the diagnosis of cardiac amyloidosis. Features of small left ventricle (LV) size with systolic impairment, increased LV wall thickening, bi-atrial enlargement, RV hypertrophy, cardiac valve thickening, and pericardial effusions are all potential findings on echo, many of which were present in our patient [8]. As the disease progresses, LV ejection fraction can slowly decrease, changing from a preserved ejection fraction to a reduced one.

Although the echocardiogram provides a useful tool in the diagnosis of the disease, it is not sufficient for diagnosis alone. It also is unable to distinguish ATTR from AL amyloidosis. For this reason, two other tests, a nuclear imaging study and an immunofixation exam, were performed in our patient. ATTR amyloidosis is confirmed with a positive predictive value and specificity of 100% if a nuclear imaging study results in a Perugini Grade 2 or 3 and immunofixation shows the absence of monoclonal proteins [13]. Because our patient’s imaging study resulted in a Perugini Grade 3 and immunofixation was negative, a diagnosis of ATTR amyloidosis could be made. However, endomyocardial biopsy remains the gold standard for diagnosis due to the ability to directly observe the deposition of amyloid fibrils within tissues.

## Conclusions

Cardiac amyloidosis is an uncommon cause of new or worsening heart failure. Diagnosis rests on a strong clinical suspicion and electrocardiographic and echocardiologic findings, along with confirmative sereolgic and imaging studies as described above. Previously endomyocardial biopsy was necessary to confirm the diagnosis, but today serologic, imaging, and fat pad biopsy can be used to establish the diagnosis with certainty.
